# TREM-1 Is Induced in Tumor Associated Macrophages by Cyclo-Oxygenase Pathway in Human Non-Small Cell Lung Cancer

**DOI:** 10.1371/journal.pone.0094241

**Published:** 2014-05-19

**Authors:** Zhihong Yuan, Hiren J. Mehta, Kamal Mohammed, Najmunissa Nasreen, Robert Roman, Mark Brantly, Ruxana T. Sadikot

**Affiliations:** 1 Veterans Affairs Medical Center, Gainesville, Florida, United States of America; 2 Division of Pulmonary, Critical Care and Sleep Medicine, University of Florida, Gainesville, Florida, United States of America; Istituto Superiore di Sanità, Italy

## Abstract

It is increasingly recognized that the tumor microenvironment plays a critical role in the initiation and progression of lung cancer. In particular interaction of cancer cells, macrophages, and inflammatory response in the tumor microenvironment has been shown to facilitate cancer cell invasion and metastasis. The specific molecular pathways in macrophages that immunoedit tumor growth are not well defined. Triggering receptor expressed on myeloid cells 1 (TREM-1) is a member of the super immunoglobulin family expressed on a select group of myeloid cells mainly monocyte/macrophages. Recent studies suggest that expression of TREM-1 in tumors may predict cancer aggressiveness and disease outcomes in liver and lung cancer however the mechanism of TREM-1 expression in the setting of cancer is not defined. In this study we demonstrate that tumor tissue from patients with non-small cell lung cancer show an increased expression of TREM-1 and PGE_2_. Immunohistochemistry and immunofluorescence confirmed that the expression of TREM-1 was selectively seen in CD68 positive macrophages. By employing an *in vitro* model we confirmed that expression of TREM-1 is increased in macrophages that are co-cultured with human lung cancer cells. Studies with COX-2 inhibitors and siCOX-2 showed that expression of TREM-1 in macrophages in tumor microenvironment is dependent on COX-2 signaling. These studies for the first time define a link between tumor COX-2 induction, PGE_2_ production and expression of TREM-1 in macrophages in tumor microenvironment and suggest that TREM-1 might be a novel target for tumor immunomodulation.

## Introduction

Lung cancer is one of the deadliest cancers worldwide. Non-small cell lung cancer (NSCLC) accounts for more than 80% of all lung cancers. On average, the 5-year survival rate for NSCLC is approximately 15% [1]. Although significant advances have been made with conventional therapies, the low overall survival and poor prognosis for patients with lung cancer indicates the need to develop new treatment options for this devastating disease [2]. As a result, there has been continued quest to define the potential pathways that drive the tumorgenesis in lung cancer with a hope to develop alternative and/or adjunctive therapies for lung cancer.

It is increasingly recognized that the tumor microenvironment plays a critical role in the initiation and progression of lung cancer. Tumor development depends on factors in the microenvironment; interactions between malignant cells, stromal cells, extracellular-matrix components, various inflammatory cells, and a range of soluble mediators contribute to tumor development and progression [3] [4] [5] [6]. Macrophages in tumors are usually referred to as tumor-associated macrophages (TAMs) and their presence can be substantial (up to 60% of the tumor stroma). A hallmark of macrophages is their plasticity, an ability to either aid or fight tumors depending on the tumor environment, which has given them the reputation of a double-edged sword in tumor biology [7] [8] [9] [10] [11] [12]. There is accumulating evidence that cancer cells can recruit and subvert macrophages to serve as active collaborators in their neoplastic program. Persistent activation of macrophages causes local chronic inflammation, production of cytokines and chemokines that promotes tumorigenesis [3] [4] [6] [9] [13] [14]. However the molecular mechanisms by which tumors activate macrophages to promote tumor growth are not well defined.

TREM proteins (Triggering receptors expressed on myeloid cells) are a family of immunoglobulin cell surface receptors expressed on myeloid cells [15]. The TREM family of protein receptors consists of TREM-1, TREM-2, TREM-3 (mouse), TREM-like transcript (TLT)-1, and TLT-2. The TREM gene cluster is located on human chromosome 6p21 and mouse chromosome 17C3 [16] [17]. TREM-1 was the first TREM identified and initial studies established TREM-1 as an amplifier of the systemic inflammatory response syndrome and sepsis [18] [19] [17] [20]. The precise ligand for TREM-1 is unknown however we and others have shown that bacterial and viral products [21] [19] induce expression of TREM-1. Additionally, we have shown that MyD88 dependent and independent pathways activate TREM-1 in response to specific TLR ligands [21]. The functional consequences of silencing TREM-1 gene in macrophages include an altered availability of key signaling (CD14, IκBα, MyD88), and effector molecules (MCP-1, IL-1β, IL-6, IL-23) downstream of TLR activation [22]. Recent studies have also shown that lipid mediators such as prostaglandins modulate expression of TREM-1. In particular PGE_2_ induces whereas PGD_2_ and PGJ_2_ inhibit the expression of TREM-1 [23] [24]. Together these studies have suggested a pivotal role for TREM-1 in amplification of TLR induced responses. However, the role of TREM-1 in tumor associated inflammation and microenvironment has not been established.

Recent studies have shown that TREM-1 is highly expressed in colon, hepatocellular and lung carcinoma tissue [25] [26] [27] [5]. Furthermore, TREM-1 expression in patients with NSCLC has been associated with cancer recurrence and poor survival of patients suggesting that TREM-1 may play an important role in cancer progression [27]. However the mechanism by which TREM-1 is induced in tumor tissue has not been defined.

We conducted this study to determine cell specific expression of TREM-1 in human non-small cell lung cancer (NSCLC) tissue and to define the mechanism by which tumor cells induce expression of TREM-1. We hypothesized that TREM-1 activation in tumor associated macrophages may be induced by PGE_2_ generated by cyclo-oxygenase-2 from tumor cells. We investigated our hypothesis using a co-culture system of human cancer cells with human monocyte/macrophages *in vitro*.

## Materials and Methods

### Ethics Statement

Human normal lung and NSCLC were obtained both in frozen form and as cell blocks from clinical and translational research institute (CTSI) at University of Florida which provides a tissue bank for research purposes. Written informed consent was obtained from all patients whose tissues have been banked. Written consents have been filed for all the study participants. The study was approved by the Ethics committee and the Institutional Review Board at University of Florida. Approval number (#IRB201200392).

### Reagents

Fetal bovine serum (FBS) (10%) was obtained from AmericanType Culture Collection (ATCC, Manassas, VA). RPMI-1640 medium was obtained from Gibco (Invitrogen Corporation, Carlsbad, CA). phorbol 12-myristate 13-acetate (PMA), NS398 were obtained from Sigma–Aldrich (St. Louis, MO). EP1, EP2, EP4, receptor antagonists (GW848687, AH6809, AH 23848) and recombinant PGE2 and PGD2 were purchased from Cayman chemicals. EP1 antagonist (L-798-106) was purchased from Tocris Bioscience. cAMP agaonist (forskolin) was purchased from Sigma-Aldrich. COX-2 and actin antibodies, non-targeting siRNA pool (NS siRNA) and siRNA targeting COX-2 were obtained from Santa Cruz Biotechnologies (Santa Cruz, CA).

### Cell Culture and Co-culture Experiments

Human monocytic cell line U937, lung cancer cell line A549, H23 or H838 were purchased from ATCC, and maintained in RPMI-1640 medium supplemented with 10% FBS. A549, H23 or H838 cells were plated into six-well dishes (1.5 to–2×10^6^ cells per well) in the culture media. U937 cells were treated with PMA (10 ng/ml) for 48 h to differentiate them into macrophages. U937 cells (2 to –2.5×10^6^ cells per insert), human macrophages (10^6^ cells per insert) were plated directly on the transwell inserts (0.4 µm, BD Biosciences) in culture medium. Prior to coculture, lung cancer cells and macrophages were washed with RPMI containing 0.1% bovine serum albumin (basal medium). After the last wash, the appropriate basal medium was added to the lung cancer cells and inserts containing macrophages were placed in each well.

### Preparation of Human Macrophages from Peripheral Blood Monocytes

Human peripheral blood monocytes (PBMCs) were isolated from buffy coats of normal donors over a Ficoll-paque PLUS (GE Healthcare) gradient. PBMCs were differentiated to macrophages by cultivation in RPMI-1640 supplemented with 10% FBS (Gibco) at a density of 10^6^/ml for seven days. For maturation of human macrophages,50 ng/ml human M-CSF (R&D systems) was supplemented into complete media. Purity of macrophages were controlled by flow cytometry (>90% CD14^+^).

### Immunohistochemistry Analysis

Lung cancer specimens and normal tissue from para carcinoma area were obtained from three patients with non-small cell lung cancer. Paraffin sections (4 µm) were stained with hematoxylin and eosin for histologic evaluation. Immunohistochemical staining was performed using a modified avidin–biotin peroxidase complex method. 4 µm sections from formalin-fixed, paraffin-embedded tissues were mounted on poly-L-lysine-coated slides and then deparaffinized. Endogenous peroxidase activity was blocked with 3% hydrogen peroxide for 15 min at room temperature. After antigen retrieval in HIER (pH 6.0) for 4 minutes, the sections were incubated overnight at 4°C with anti-TREM-1 antibody (HPA005563, Sigma-Aldrich) or isotype matched control antibody at the dilution of 1∶500, then detected using EnVision™ System-HRP (DAB)(Dako). The specificity of immunohistochemistry was verified using an antibody isotype control replacing the primary antiserum with an identical concentration of nonimmunized mouse or rabbit serum. Sections were screened at ×100 magnification to identify the regions of greatest numbers of TREM-1–positive cells. Immunostaining of TREM-1 was analyzed blindly by two board certified pathologists. Cells were counted at a density at ×400 magnification in at least three regions with the greatest numbers of TREM-1–positive cells.

### Indirect Immunofluorescence

Sections from formalin-fixed, paraffin-embedded lung cancer tissues and tumor-adjacent normal tissue were deparaffinized, then blocked with 2% horse serum in PBST buffer for 1 hr at room temperature. After blocking, slides were incubated with anti-TREM-1 antibody(HPA005563, Sigma) at the dilution of 1∶500 overnight at 4°C, after three washes in cold PBS, blotted with secondary antibody (Alexa Fluor 594 donkey anti-rabbit IgG,1∶500; Molecular Probes) for 1 hr at room temperature. For dual staining, slides were washed again, with blocking solution, and incubated with anti-human CD68 antibody(clone KP1, 1∶100; Abcam) and another fluoro-conjugated secondary antibody (Alexa Fluor 488 donkey anti-mouse IgG,1∶500; Molecular Probes). DAPI-containing mounting medium (Molecular Probes) was used for nuclear staining. Images at a single cell level were observed and photographed using a fluorescence microscope equipped with a Charge Couple Device camera (Leica SP5, Germany).

### RNA Interference

Cells were transfected with siRNA oligonucleotides targeting human COX-2 mRNA, and a non-related control siRNA (Santa Cruz) through specific LONZA transfection reagents (LONZA) according to the manufacturer’s instructions. Cells were incubated with siRNA complexes for 24 h–72 h before analysis.

### Flow Cytometry

Human macrophages and U937-differentiated macrophages were examined for receptor expression levels using FACS with anti-human CD14 APC/Cy7 (eBioscience) and anti-human TREM-1 (R&D systems). Isotype-matched monoclonal antibodies (eBioscience) were used as negative controls. Data are represented as relative percentages to control.

### Western Blotting

Immunoblotting for detection of COX-2 and actin as carried out as described previously [28].

### Reverse Transcription Polymerase Chain Reaction (RT-PCR)

Total RNA from cell lysates was isolated using the RNeasy Mini kit (QIAGEN). RNA (1 µg) was reverse transcribed using murine leukemia virus reverse transcriptase and oligo d(T)16 primer. PCR primers for human COX-2 were forward, 5′- CCCTTGGGTGTCAAAGGTAA-3′ and reverse, 5′-GCCCTCGCTTATGATCTGTC-3′. Primers for human TREM-1 were forward, 5′- GGCCACACCAACCTT CTG - 3′ and reverse, 5′-AGTGCCTGCCTC AATGTCTCCA-3′. Primers for actin were forward, 5′-AGAAAATCTGGCACCACACC-3′ and reverse, 5′-AGAGGCGTACAGGGATAGCA-3′. For real-time PCR reaction, human TREM-1 and actin primers, as well as Taqman Universal Master Mix II were obtained from Applied Biosystems, and all PCR analyses were performed on an ABI Prism 7900HT. The messenger RNA (mRNA) levels were normalized to actin. Relative expression was determined by CT (relative quantification) analysis.

### ELISA

Differentiated macrophages were stimulated with appropriate concentrations of stimulus for 12 hrs. Supernatants of cultural medium were tested for the presence of PGE_2_ using a commercially available ELISA (R&D Systems).

### Statistical Analysis

Student’s t-tests were performed to determine statistically significant differences between groups using GraphPad Prism (GraphPad Software, CA, USA). Mean levels of TREM-1, COX-2 mRNA, PGE_2_ concentration were compared utilizing analysis of variance. P-value<0.05 was considered significant.

## Results

### TREM-1 is Upregulated in Tumor Associated Macrophages in Human Non-small Cell Lung Cancer

**Figure 1:**
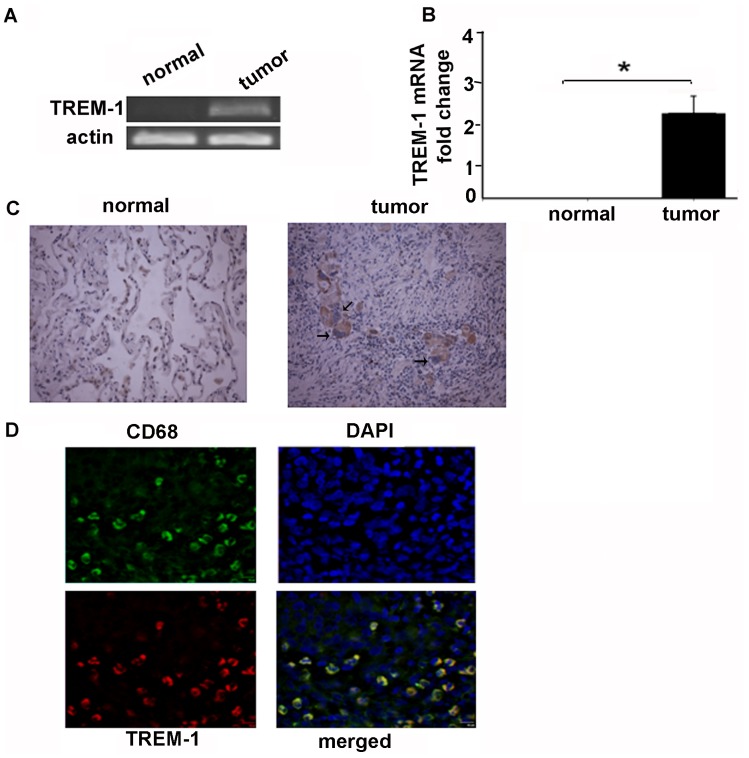
TREM-1 is upregulated in lung cancer tissue and tumor associated macrophages in human non-small cell lung carcinoma. A) RNA extracted from human lung cancer samples showed an increase in TREM-1 message. B) Real time RT PCR confirmed the increase in TREM-1 message (n = 3, *p<0.05). C) Representative immunohistochemistry image demonstrating an increased expression of TREM-1 protein in macrophages in tumor stroma D) Confocal microscopy with CD68 a specific macrophage marker and TREM-1 confirmed that cells expressing TREM are TAMs.

It is established that TREM-1 is upregulated in infection and inflammatory tissues. Since chronic inflammation is closely linked to cancer we questioned if TREM-1 is expressed in NSCLC tissue. We first performed RT-PCR and RT-qPCR from lung tissue of patients with NSCLC. We were able to detect an increase in TREM-1 message from human lung adenocarcinoma tissue whereas the expression was not detected in normal lung tissue (**Figure 1A and B**). Since the expression of TREM-1 is restricted to myeloid cells we questioned which cell types express TREM-1 in lung cancer tissue. In order to determine the specific cell types that express TREM-1 in tumor microenvironment we performed immunohistochemical analysis (using modified avidin–biotin peroxidase complex method, primary antibody anti–TREM-1 antibody and isotype-matched control antibody) from human lung cancer samples from patients with lung adenocarcinoma. Immunohistochemistry and immunoflorescent staining from the lung tumors demonstrated that TREM-1–positive cells are tumor associated macrophages (**Figure 1C**). TREM-1 positive cells are macrophages was confirmed by using CD68 staining a specific macrophage marker which confirmed that the cells are indeed macrophages (**Figure 1D**). In addition we also determined if lung cancer cells (A549 cells) express TREM-1 protein. We were not able to detect the expression of TREM-1 message or protein in tumor cells alone (data not shown). Collectively, these data suggest that TREM-1 expression is upregulated in tumor associated macrophages in non-small cell human lung cancer.

### TREM-1 is Induced in Human Macrophages Co-cultured with Lung Adenocarcinoma Cells

**Figure 2:**
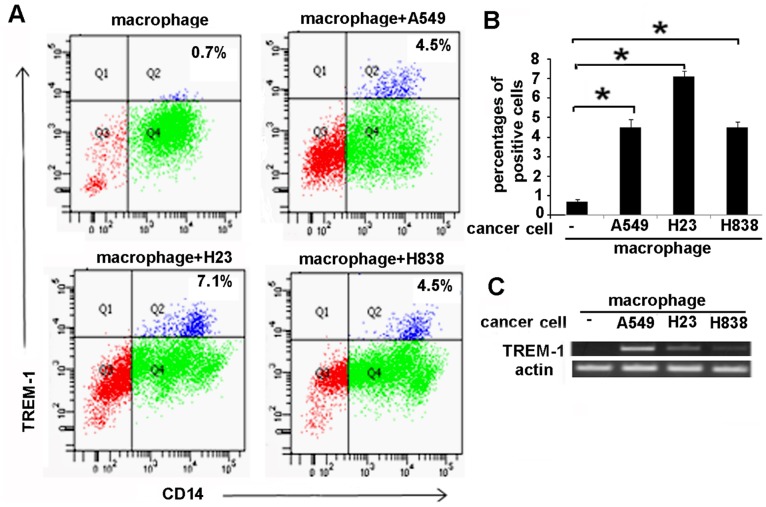
TREM-1 is induced in human macrophages co-cultured with lung adenocarcinoma cells. A) Macrophages were co-cultured with NL-20 (normal epithelial cells) or lung cancer cells (A549, H23 or H838) for 48 hours. TREM-1 expression was increased in macrophages that were co-cultured with cancer cells as demonstrated by FACS analysis B) percentage of cells that stained positive for TREM-1, n = 3–4, * p<0.05) C) Representative RT-PCR from macrophage co-cultured with tumor cells showed an showed an increased expression of TREM-1 message.

Since our data demonstrates that TREM-1 is expressed in tumor associated macrophages in human lung adenocarcinoma we developed an *in vitro* co-culture model to determine if the expression of TREM-1 in TAM in the tumor is specifically related to the tumor cells. Co-culture experiments were performed by using human macrophages (human monocytes from blood were matured to macrophages as described in methods) and A549, H23 and H838 human lung cancer cells. For these experiments tumor cells or normal epithelial cells (NL-20, ATCC) were co-cultured with matured human macrophages for 48 hours. Expression of TREM-1 was determined by FACS analysis. Co-culture of tumor cells with macrophages led to an induction of TREM-1 as determined FACS analysis (**Figure 2A**). On the other hand macrophages that were co-cultured with normal epithelial cells did not show an increase in the expression of TREM-1 protein (**Figure 2A and B**) suggesting that the expression of TREM-1 in TAMs is an effect of tumor cells on macrophages. We also performed additional experiments to confirm the increased expression of TREM-1 message. Normal epithelial cells, and cancer cells A549, H23 and H838 cells were co-cultured with human macrophages for 48 hours following which RNA was extracted from the macrophages. TREM-1 message was detected by RT-PCR. In agreement with our data with TREM-1 protein expression macrophages that were co-cultured with cancer cells showed an increase in TREM-1 message which was not detected in macrophages that were cultured with normal epithelial cells (**Figure 2C**). Together, these data confirm that tumor cells can induce expression of TREM-1 message and protein in macrophages.

### Induction of TREM-1 in Tumor Associated Macrophages is Dependent on COX-2

**Figure 3:**
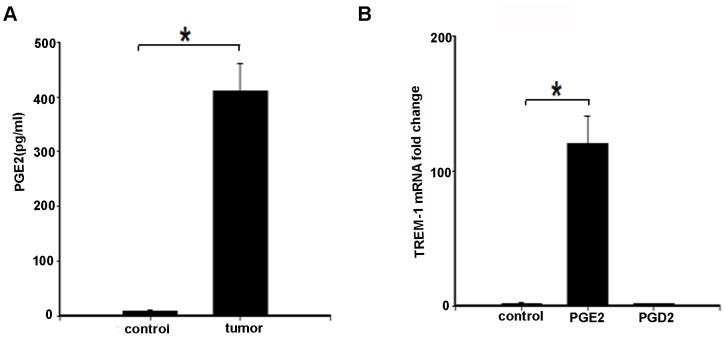
PGE_2_ is increased in human lung tumor tissue and PGE_2_ increases the expression of TREM-1 in macrophages. A) PGE_2_ levels measured from the human lung tumor tissue showed an increase in PGE_2_ message from cancer tissue with no significant detection in the normal lung (n = 3, p<0.05). B) Bone marrow derived macrophages from wild type mice were treated with recombinant PGE_2_ or PGD_2_ (10 µmol) for 12 hours and TREM-1 expression was detected. BMDM treated with PGE_2_ showed an increase in TREM-1 message in response to PGE_2_ treatment (n = 3–4, p<0.05) however TREM-1 message was not detected in cells that were treated with PGD_2_.

We next wanted to investigate the mechanism by which tumor cells induce the expression of TREM-1 in macrophages. Elevated cyclooygenase-2 (COX-2) expression has been frequently observed in human non-small cell lung cancer [10], [29]. We and others have shown that prostaglandins modulate the expression of TREM-1 in response to LPS, in particular PGE2 induces whereas PGD_2_ and PGJ_2_ inhibit the expression of TREM-1 [23] [24]. We therefore hypothesized that expression of TREM-1 in TAMS might be induced by PGE_2_ production from tumor cells via cyclo-oxygenase pathway. We first confirmed that tumor tissue and cells express COX-2 (data not shown). The levels of PGE_2_ were increased in lung tumor tissue (**Figure** **3A**). Next we treated bone marrow derived macrophages from wild type mice with recombinant PGE_2_ and PGD_2_ (10 µmol) and performed RT-qPCR to determine the expression of TREM-1 message. Treatment with PGE_2_ resulted in expression of PGE_2_ whereas cells treated with vehicle and PGD_2_ did not show induction of TREM-1(**Figure 3B**).

**Figure 4:**
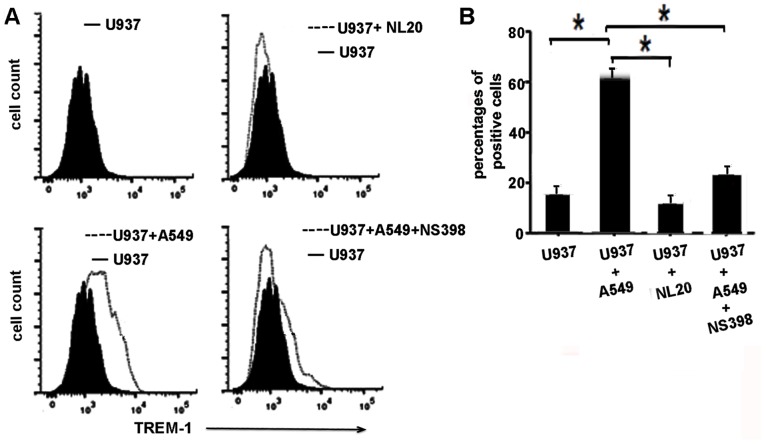
TREM-1 expression is attenuated in monocytes co-cultured with tumor cells that are treated with COX-2 inhibitors. A) Representative image of FACS analysis-U937 monocytes were co-ultured with NL-20 or A549 cells in the presence of absence of NS-398 (100 µmol) (specific COX-2 inhibitor). The expression of TREM-1 was increased in U937 cells co-cultured with A549 cells. Treatment with NS-398 inhibited this increased expression B) Percentage cells that stained positive with TREM-1 (n = 4–5, p<0.5).

**Figure 5:**
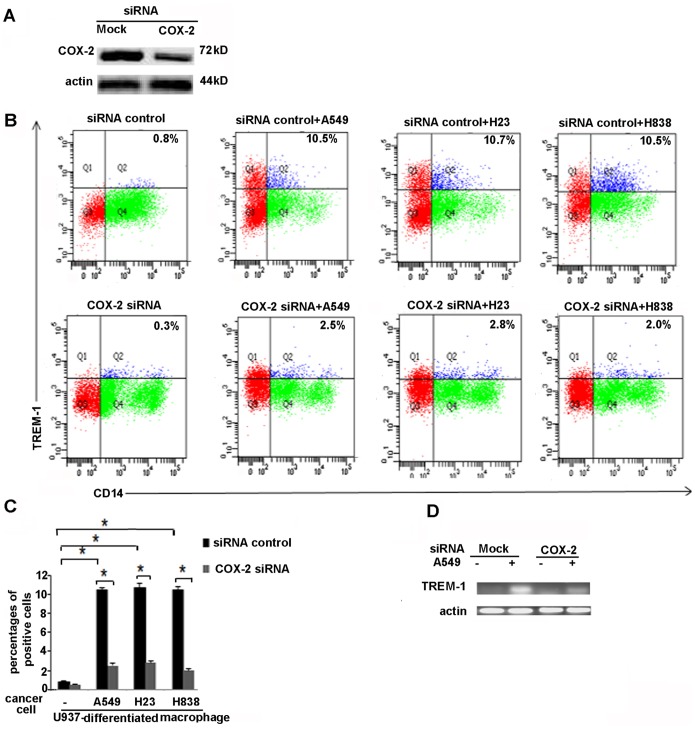
TREM-1 expression is abrogated in macrophages with COX-2 siRNA. A) Western blot analysis from human macrophages with COX-2 siRNA confirmed knock down of COX-2 protein in cells with siCOX-2 compared to the mock (control) siRNA. B) FACS images demonstrating TREM-1 expression from human macrophages expressing control siRNA or COX-2 siRNA co-cultured with NL-20 cells or cancer cells (A549, H23 or H 838 cells). The expression of TREM-1 was increased in macrophages expressing control siRNA co-cultured with cancer cells whereas macrophages with siCOX-2 showed an attenuated expression of TREM-1. C) Percentage of cells that stained positive for TREM-1 staining n = 3, p<0.05). D) Representative RT-PCR from macrophages with siCOX-2 show a decreased TREM-1 message compared to macrophages with control siRNA.

In order to confirm the role of COX-2 in induction of TREM-1 we performed experiments where we co-cultured lung cancer cells with monocyte/macrophages for 48 hours in the presence of COX-2 inhibitors or vehicle treatment. Control macrophages were co-cultured with normal epithelial cells NL-20. Expression of TREM-1 was determined by FACS analysis. As shown in Fig 4A monocytes (U937) cells that were co-cultured with cancer cells (A549 cells) showed an increased expression of TREM-1 protein which was not detected when U937 cells were co-cultured with NL-20 normal epithelial cells. Treatment with COX-2 inhibitor (NS-398, 100 µ mol) resulted in abrogation of the induction of TREM-1 (**Figure 4A and 4B**). In order to conclusively define the role of COX-2 induction of TREM-1 we knocked down COX-2 expression in macrophages by employing siCOX-2. Macrophages were transfected with siCOX-2 or control siRNA for 48 hours prior to co-culturing with cancer cells or normal epithelial cells (NL20) and expression of TREM-1 protein was detected by FACS analysis. We first confirmed that expression of COX-2 was attenuated in the COX-2 knockdown cells as determined by western blot analysis for COX-2 (**Figure 5A**). As shown in figure 5B and C TREM-1 protein was upregulated in macrophages expressing control siRNA that were co-cultured with lung cancer cells (A549, H23 and H838 cells) but not in macrophages that were cultured with normal epithelial cells. However the expression of TREM-1 protein was abrogated in macrophages transfected with COX-2 siRNA that were co-cultured with cancer cells (A549, H23 or H838 cells) (**Figure 5B, 5C** **).** Expression of TREM-1 message was also abrogated in macrophages that were treated with siCOX-2 and co-cultured with tumor cells (**Figure 5D**). Together these data conclusively show that expression of TREM-1 is COX-2 dependent and is mediated by the production of PGE_2_.

**Figure 6:**
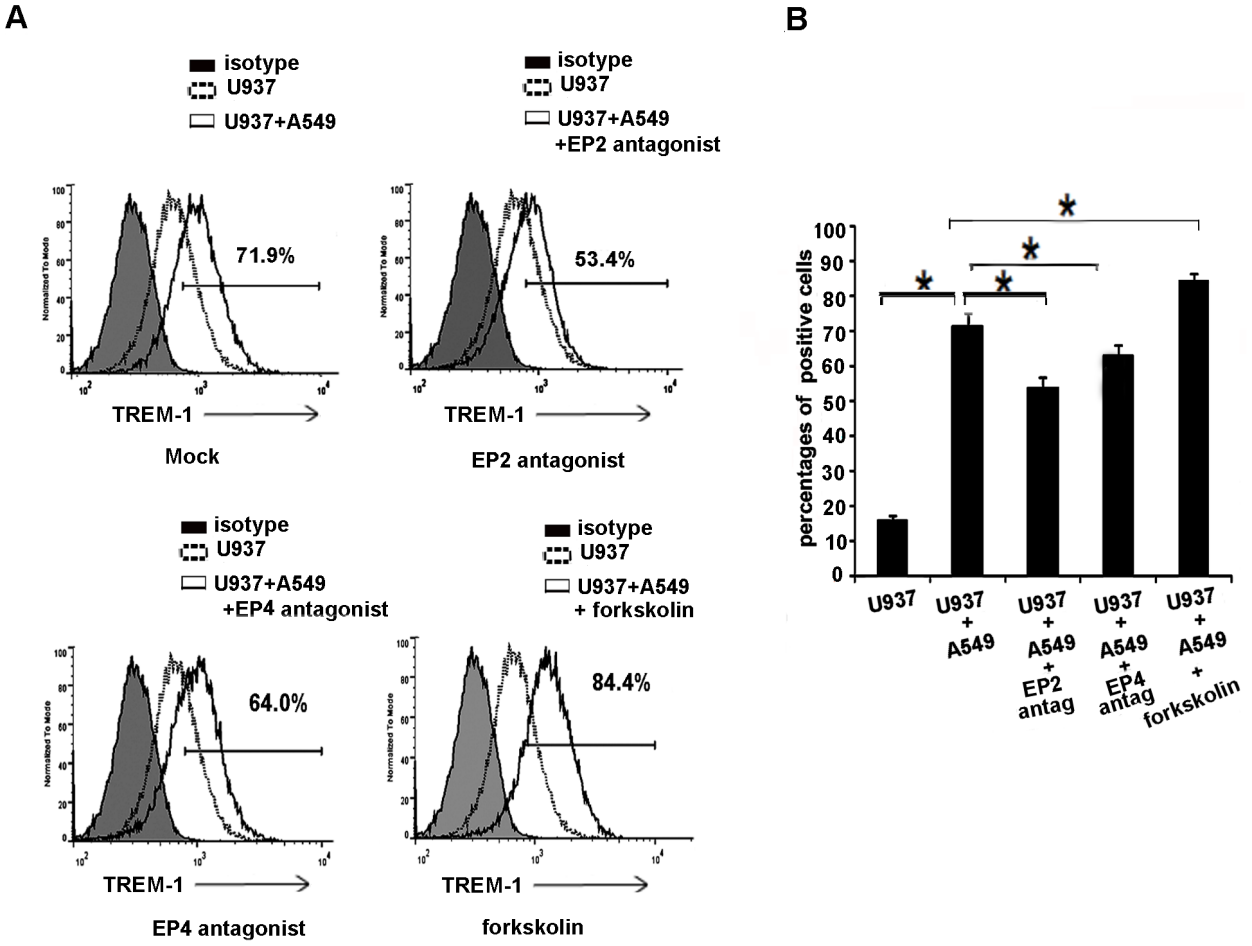
A) FACS images demonstrating that TREM-1 expression is attenuated in macrophages that are co-cultured with tumor cells in the presence of EP2 and EP4 antagonist (AH6809 and AH23848) whereas it is increased in the presence of forskolin. B) Percentage cells that stained positive with TREM-1 (n = 4–5, *p<0.05).

We next wanted to define the mechanism by which PGE_2_ mediates the expression of TREM-1 in tumor associated macrophages. PGE_2_ influences cell behavior through the ligation of its four distinct G-protein-coupled E-prostanoid receptors, numbered EP1–4 [30], [31]. In order to determine the receptors through which PGE_2_ alters the expression of TREM-1 we performed experiments with the EP1 through EP4 antagonists. Macrophages were treated with the respective antagonist (10, 50 µmol) prior to co-culturing with A549 cells. EP1 (GW848687) and EP3 antagonists (L-978-106) had no significant effect on the expression of TREM-1 (data not shown), however cells that were treated with EP2 (AH6809) and EP4 (AH23848) antagonist showed a reduced expression of TREM-1 protein as determined by FACS analysis (**Fig 6A and B**). EP2 and EP4 receptors are potent immunoregulatory molecules that share the capacity to increase intracellular concentrations of cyclic adenosine monophosphate (cAMP) within seconds to minutes of PGE_2_ binding. Thus we wanted to determine if these effects are mediated through increase in cAMP levels. We therefore performed experiments with cAMP agonist forskolin (10 µmol) and in agreement with our data with EP2 blockade we found a significant increase in the expression of TREM-1 in response to forskolin (**Fig 6A and 6B**). Collectively these data show that the expression of TREM-1 in tumor associated macrophages which is induced by PGE_2_ is mediated through EP2 and EP4 receptors and is driven by an increase in the level of cAMP.

## Discussion

Our study convincingly shows that TREM-1 is expressed in human NSCLC tissue and that the expression is selectively seen in the tumor associated macrophages in the cancer stroma. We were not able to detect the expression of TREM-1 in normal lung tissue or in isolated tumor cells. Additionally we show that tumor tissue has an increased expression of COX-2 with PGE_2_ production and that macrophages that are treated with PGE_2_ show an increased expression of TREM-1. Furthermore treatment with COX-2 inhibitors and knockdown of COX-2 in macrophages attenuated the expression of TREM-1 in a co-culture model of lung cancer cells with macrophages. Together these data suggest that TREM-1 may be a critical link in the tumor microenvironment between tumor-associated macrophage activation, inflammatory response, and cancer progression.

Cancer-related inflammation is now recognized as a hallmark of tumors and the link between lung carcinogenesis and chronic immune activation is well established [32] [33] [34] [35] [36]. The inflammation present in tumor microenvironment is characterized by leukocyte infiltration which includes tumor-associated macrophages mast cells, dendritic cells, natural killer cells, neutrophils, eosinophils and lymphocytes [37] [38] [32]. It is also increasingly recognized that interaction of cancer cells, macrophages, and inflammatory response in the tumor microenvironment may facilitate cancer cell invasion and metastasis [39] [40] [41] [42] [43] [44]. Previously, we have shown that resident lung macrophages are crucial effectors of susceptibility to metastatic lung cancer growth [45]. Several mouse and human studies have shown that high TAM density is mostly associated with poor patient prognosis and resistance to therapies in a variety of cancers including non-small cell lung cancer [46] [47] [40] [41] [42] [48] [44] [49]. Furthermore, monocyte/macrophage depletion in experimental settings has been successful in limiting tumor growth and metastatic spread and in achieving better responses to conventional chemotherapy and antiangiogenic therapy [50] [13]. Although TAMs contribute significantly to production of VEGF, IL-1β, TNF-α, IL-6, IL-23, IL-8, MMPs which have pro-angiogenic and growth attributes, specific molecular pathways in macrophages that immunoedit tumor growth are not well defined [37]. We found abundant macrophages in the stroma of the tumor tissue which showed both TREM-1 and CD68 expressions. CD68 is a macrophage marker for tumor-associated macrophage that plays an important role in angiogenesis and metastasis. Increase in CD68 positive macrophages in our study supports the hypothesis that inflammatory macrophages expressing TREM-1 may contribute to continued production of mediators which may promote tumor growth.

Triggering receptor expressed on myeloid cells 1 (TREM-1) is a member of the super immunoglobulin family expressed on a select group of myeloid cells mainly monocyte/macrophages. Expression of TREM-1 has been associated with immune inflammatory response. Inflammation promotes multiple hallmarks of cancer, such as sustained proliferative signaling, resistance to cell death and induction of angiogenesis [51] [35] [36]. Recent studies suggest that expression of TREM-1 in tumors may predict cancer aggressiveness and disease outcomes in liver and lung cancer implicating that expression of TREM-1 in macrophages may be a associated with tumor growth and progression [25] [26] [27]. We have shown that the functional consequences of silencing TREM-1 gene in macrophages include an altered availability of key signaling (CD14, IκBα, MyD88), and effector molecules (MCP-1, IL-1β, IL-6, IL-23) downstream of TLR activation [22] [21] [19]. In particular production of IL-6 and IL-23 is exaggerated by TREM-1 activation [22], [52]. IL-6 and IL-23 have been shown to be involved in immuno-editing in carcinoma microenvironments and is associated with poor prognosis [53] [54] [55] [56]. Although speculative the exaggerated production of these mediators by TREM-1 may be a mechanism by which TREM-1 expression in TAMS can promote tumor growth and progression. Ongoing and future studies will establish the role of TREM-1 in tumor growth and define the mechanisms by which TREM-1 modulates tumor growth [27] [56].

**Figure 7:**
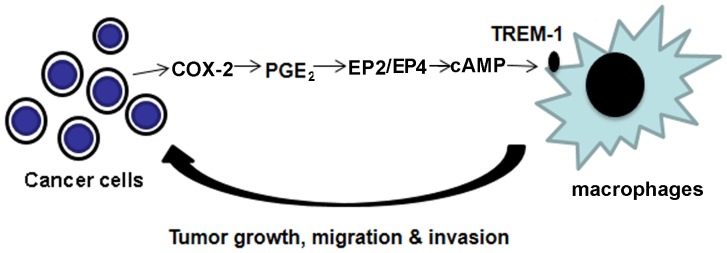
Proposed mechanism of TREM-1 expression in TAMs by lung cancer cells. COX-2 induction in lung cancer cells leads to production of PGE2 which then causes expression of TREM-1 in tumor associated macrophages. These effects are medicated through EP2 and EP4 receptors and driven through cAMP. Expression of TREM-1 in macrophages increases production of mediators that propagate tumor growth, migration and invasion.

In this study we also investigated the mechanism by which TREM-1 is expressed in TAMS. Since the expression of TREM-1 is modulated by lipid mediators particularly prostaglandin we investigated the role of cyclo-oxygenase pathway. We show that inhibition of COX-2 led to attenuation of expression of TREM-1 in macrophages. To our knowledge this is the first study that demonstrates that expression of TREM-1in TAMS in tumor microenvironment is dependent on the COX-2 signaling pathway. We also show that lung tumor tissue has an increased production of PGE_2_ which probably contributes to the expression of TREM-1 in TAMs. Furthermore our studies with the EP receptor antagonists show that the effects of PGE2 in tumor micro-environment are mediated by EP2 and EP4 receptor and are a result of increase in cAMP. Future studies will define the detailed mechanisms and signaling pathways [31] employed by PGE_2_ in tumor microenvironment which are responsible for the expression of TREM-1 in TAMs. High expression of COX-2 in NSCLC cells is associated with tumor promotion, invasion and metastasis and is associated with poor prognosis [29] [57]. Furthermore inhibition of COX-2 is associated with reduced risk of developing lung cancer in animal models and in smokers [58] [59] [60] [61]. The mechanism by which COX-2 promotes lung tumorigenesis is not fully understood. Our data suggest that expression of COX-2 in tumor cells leads to production of PGE2 which through EP2 and EP4 receptors leads to induction of TREM-1 (**Figure 7**). These data provide important clues to the link between COX-2 induced PGE_2_ production with TREM-1 expression and perhaps tumor progression.

In summary this study shows that human non-small cell lung cancers have a high expression of TREM-1 in tumor associated macrophages. The expression of TREM-1 in tumor microenvironment is dependent on the cyclo-oxygenase pathway and is mediated by increased production of PGE_2_.by tumor cells. To our knowledge this is the first study to define a link between cyclooxygenase pathways in non-small cell lung cancer with TREM-1 signaling. Understanding the link between these key inflammatory pathways is of fundamental significance in defining tumor immune response and developing immunotherapies for lung cancer. Our study suggests that TREM-1 inhibition may prove to be an adjunctive therapy to limit tumor growth. Further studies to define the role of TREM-1 in tumor immunomodulation in tumor microenvironment are needed.
